# Potential Antigenic Candidates for the Development of Peptide-Based Vaccines to Induce Immunization against *Helicobacter pylori* Infection in BALB/c Mice

**DOI:** 10.3390/ijms232112824

**Published:** 2022-10-24

**Authors:** Doaa M. AlEraky, Hatem M. Abuohashish, Amr S. Bugshan, Maha M. Abdelsalam, Hussain A. AlHawaj, Taleb T. AlKhamis, Fatimah A. AlDossary, Nabras M. Alrayes, Yasser M. Ragab, Zeinab AbdelKhalek, Omneya M. Helmy, Mohammed A. Ramadan

**Affiliations:** 1 Department of Biomedical Dental Science, College of Dentistry, Imam Abdulrahman Bin Faisal University, P.O. Box 1982, Dammam 31441, Saudi Arabia; 2 Institute for Research and Medical Consultations, Imam Abdulrahman Bin Faisal University, P.O. Box 1982, Dammam 31441, Saudi Arabia; 3 College of Dentistry, Imam Abdulrahman Bin Faisal University, P.O. Box 1982, Dammam 31441, Saudi Arabia; 4 Department of Microbiology and Immunology, Faculty of Pharmacy, Cairo University, Cairo 11562, Egypt; 5 Department of Medical Microbiology and Immunology, Faculty of Medicine, Cairo University, Cairo 11562, Egypt

**Keywords:** *H. pylori*, vaccinology, cellular immunity, humoral immunity, immunoinformatic analysis, immunization, interleukins, immunoglobulins, stomach, histopathology

## Abstract

*Helicobacter pylori* (*H. pylori*) has been identified as a group-1 definite carcinogen. As of yet, there is no available vaccine for this microorganism. Our study aimed to identify antigenic peptides in *H. pylori* using an in silico proteomic approach, and to evaluate their effectiveness as potential vaccine candidates. Four different peptide sequences were prioritized using the reverse vaccinology, namely, CagA^1^, CagA^2^, VacA, and SabA. Peptides emulsified with Freunde’s adjuvant were used to immunize BALB/C mice. Subcutaneously immunized mice were challenged by oral administration of *H. pylori.* IgG, IgA, IL4, and IL17 were detected in mice sera. Histopathology of the dissected stomach of vaccinated and control mice were assessed using H&E stain. IgG was significantly higher in mice vaccinated with SabA. IL-4 was significantly increased in CagA^1^, CagA^2^, VacA, and SabA vaccinated mice compared to the adjuvant group. Additionally, histopathological examination of gastric tissue showed a protective effect in the vaccinated groups compared to adjuvant and PBS groups. Our findings indicate a promising effect of the tested epitopes, particularly the SabA antigen, to induce an immune response against *H. pylori*.

## 1. Introduction

*Helicobacter pylori* (*H. pylori*) colonizes the gastric mucosa of around 50% of the human population, with the highest prevalence in the developing countries [1]. It is the leading cause of upper gastrointestinal diseases, including chronic gastritis and, consequently, peptic ulcer disease, as well as, to a less extent, gastric mucosa-associated lymphoid tissue lymphoma and gastric carcinoma [2,3,4]. *H. pylori* infection is hyper-endemic in various age groups of patients, including individuals with non-ulcer dyspepsia [5,6]. The eradication treatment of *H. pylori* is becoming less successful, with a reported increase in resistance to previously efficient antibiotic regimens [7,8]. Owing to the carcinogenic nature of *H. pylori* and its potential long-term presence from childhood in the gastrointestinal tract, vaccination recruitment is considered a necessary and alternative approach, or a complementary method to antibiotic therapy to control *H. pylori* infection [9]. Reverse vaccinology has revolutionized vaccine development, where potential vaccine candidates are selected from the pathogen’s genome/proteome and prioritized [10,11,12]. Yet, the absence of a protective vaccine increases the worldwide risk of infection. Several attempts to develop vaccines for *H. pylori* have been reported [13]. Among these, a trivalent vaccine, administrated by the intramuscular route, comprising three proteins (VacA, CagA, and neutrophil activating protein) was reported. Although recognized by both the host’s humoral and cellular immune systems, no immunity was detected in the challenged model [14,15,16]. Cytotoxin-associated gene A (CagA) and vacuolating cytotoxin A (VacA) are among the major virulence factors of *H. pylori* that play a pivotal role in inducing gastric cancer and peptic ulcer [17,18,19]. Moreover, Sialic acid-binding adhesin (SabA) is one of the most important adhesions that attaches to the inflamed gastric mucosa. Moreover, it can adhere to erythrocytes in the capillaries of infected gastric mucosa, and leach from the lumen to disseminate into the circulation [20]. A study by Malfertheiner et al. reported satisfactory safety and immunogenicity of CagA and VacA using aluminum hydroxide as an adjuvant. However, in a clinical trial study, compared with placebo, the vaccine did not confer additional protection against *H. pylori* infection after being challenged with a CagA-positive strain [21,22]. In 2021, a study has characterized the effect of multi-epitope vaccine based on Melittin as an adjuvant, using Western blotting analysis. Yet, further research was recommended to investigate the efficiency of the designed vaccine against *H. pylori* [23].

The immune responses involved in *H. pylori* infection are highly complex, with several ambiguous aspects. IgG and IgA are common antibodies in different body fluids, and they shield against multiple bacterial and viral infections. In serum, the IgG level is likely to increase with *H. pylori* infection, and it was suggested as a diagnostic tool for this infection [24]. Unlike IgG, IgA can be transferred into the gastric lumen, and is considered the initial defense line against *H. pylori* infection [25]. Studies have shown that the pro-inflammatory IL-17 plays a vital role in the pathogenesis of *H. pylori* infection, which promotes IL-17 expression in human gastric mucosa, leading to attraction of inflammatory neutrophils and resulting in bacterial persistence [26]. Recent studies have found that IL-17 levels were increased in patients with *H. pylori* infection and positively correlated with chronic gastritis severity [27,28]. The IL-4 is a multi-functional anti-inflammatory cytokine, which represses the pro-inflammatory pathological environment. Chatterjee et al. reported that *H. pylori* infection triggers the production of IL-4 from peripheral blood lymphocytes and gastric tissues in human subjects with or without *H. pylori* [29]. Another study observed that human gastric epithelium cells produce IL-4 in response to *H. pylori* infection [30]. On the other hand, the increased IL-4 production in the study conducted by Fan et al. did not suppress proliferation of peripheral blood lymphocytes in *H. pylori* infection [31]. Another study reported that IL-4 suppressed the production of inflammatory cytokines, such as tumor necrosis factor-α and IL-1β, by the gastric mononuclear cells in patients with *H. pylori* infection [32]. 

Accordingly, there is a need for radical approaches to face the failures in previous *H. pylori* vaccine studies [33,34]. Our study aims to use immunoinformatic tools to identify potential vaccine candidates in *H. pylori* and to evaluate their prophylactic effect in a BALB/c mice animal model.

## 2. Results

**Figure 1:**
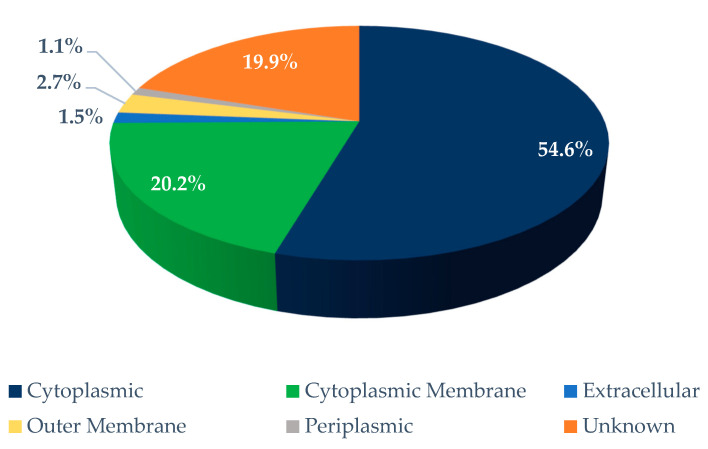
The results of subcellular localization of the predicted proteins of *H. pylori* strain 266595 by the protein localization prediction PSORTb server. Out of a total of 1445 proteins, the exact data reported at the PSORTdb server were 766 cytoplasmic (54.6%), 284 cytoplasmic membrane (20.2%), 21 extracellular (1.5%), 38 outer membrane (2.7%), 15 periplasmic membrane (1.1%), and 280 unknown proteins (19.9%). The predicted extracellular and outer membrane proteins were included in the prioritized dataset.

The *H. pylori* strain 266595 (NC_000915.1) is composed of 1.6 Mb and 1442 coding genes. The subcellular localization prediction revealed 38 outer membrane and 21 extracellular proteins successively, according to the scores of other parameters https://db.psort.org/browse/genome?refseq=NC_000915 (accessed on 31 May 2020) (Figure 1).

A total of seven proteins were selected according to the ranking criteria, including subcellular localization, virulence, non-human homology, TM helixes, and molecular weight. Proteins with antigenic and pathogenic mechanisms were CagA, OipA, and VacA. Additionally, FlaA, FecA, BabA, and SabA were involved in the processes of adhesion and colonization. Extracellular proteins were CagA and FlaA. However, OipA, FecA, SabA, VacA, and BabA were predicted as outer membrane proteins. Homology analysis of the prioritized proteins using BLASTp demonstrated the sequences as non-human homologs. In the prediction of the topology of proteins by TMHMM, VacA showed the presence of one helix, whereas other proteins did not unveil any such topology. Molecular weights of the selected proteins resulted in weight < 110 kDa, except for VacA and CagA. One critical characteristic of a good vaccine candidate is that it should not present a homology with human proteins to avoid any potential autoimmune response. Candidates should also possess good antigenic properties, which are essential for the pathogenesis of the microorganism and protection against the disease (Supplementary Tables S1 and S2).

Finally, three antigenic, virulent, and non-homologous proteins (CagA, VacA, and SabA) were prioritized and four potential epitopes were designated: CagA^1^, CagA^2^, VacA, and SabA, as listed in Table 1.

**Figure 2:**
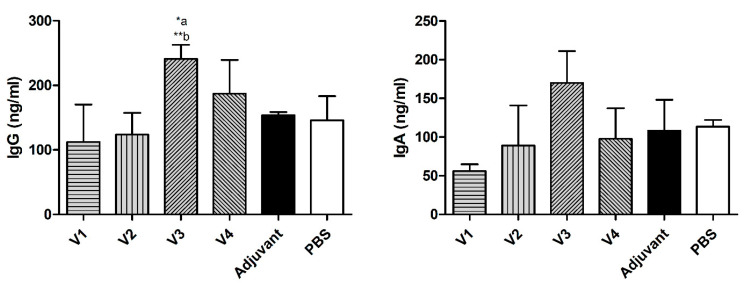
Effects of different vaccines (V1, V2, V3, and V4) on serum levels of immunoglobulin-G (IgG) and immunoglobulin-A (IgA) in BALB/c mice challenged with *H. pylori*. Data are presented as mean ± SD (*n* = 6) and statistically analyzed by one way ANOVA, followed by the Tukey–Kramer post hoc test. Statistical significance was considered when * *p* ≤ 0.05 and ** *p* ≤ 0.01. “a” vs. PBS group and “b” vs. adjuvant group.

IgG levels in the serum samples from the V3 group were significantly higher than those in the PBS group (*p* ≤ 0.05) and the adjuvant group (*p* ≤ 0.01). In addition, IgA levels in the serum samples from all groups showed no significant difference between the groups, although they were higher in the V3 group (Figure 2).

**Figure 3:**
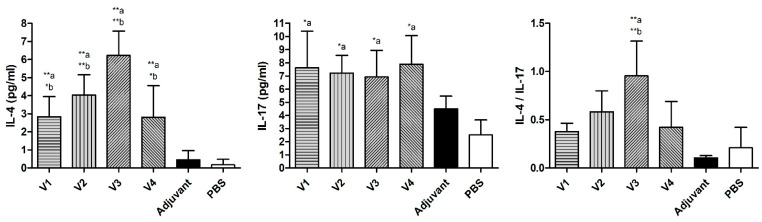
Effects of different vaccines (V1, V2, V3, and V4) on serum levels of interleukin-4 (IL-4), interleukin-17 (IL-17), and their ratio in BALB/c mice challenged with *H. pylori*. Data are presented as mean ± SD (*n* = 6) and statistically analyzed by one way ANOVA, followed by the Tukey–Kramer post hoc test. Statistical significance was considered when * *p* ≤ 0.05 and ** *p* ≤ 0.01. “a” vs. PBS group and “b” vs. adjuvant group.

Serum levels of IL-4 were significantly higher in groups V1 (*p* ≤ 0.01), V2 (*p* ≤ 0.01), V3 (*p* ≤ 0.01), and V4 (*p* ≤ 0.01), compared to the PBS group. IL-4 serum levels were also significantly higher in V1 (*p* ≤ 0.05), V2 (*p* ≤ 0.01), V3 (*p* ≤ 0.01), and V4 (*p* ≤ 0.05) groups, compared to the adjuvant group. In the case of serum levels of IL-17, there was a significant increase in its levels in groups V1 (*p* ≤ 0.05), V2 (*p* ≤ 0.05), V3 (*p* ≤ 0.05), and V4 (*p* ≤ 0.05), compared to the PBS group. The IL-4/IL-17 ratio was significantly high only in the V3 group as compared with the PBS (*p* ≤ 0.01) and adjuvant (*p* ≤ 0.01) groups (Figure 3).

**Figure 4:**
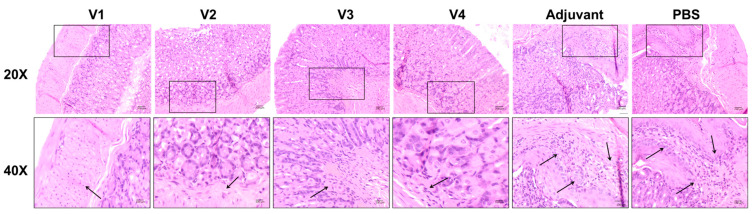
Photomicrographs of hematoxylin and eosin (H&E)-stained sections of stomach and proximal duodenum of mice challenged with *H. pylori*. Images were taken at 20× (non-bordered images) and 40× (black bordered images) magnifications. Histopathology of stomach tissues from the vaccinated groups (V1, V2, V3, and V4) exhibited less inflammation to patchy infiltration of mixed leukocytes in the mucosa and submucosa (black arrows). Histopathology of gastric tissues of the adjuvant and PBS groups showed a marked increase in leukocyte infiltration extending below the submucosa (black arrows).

**Figure 5:**
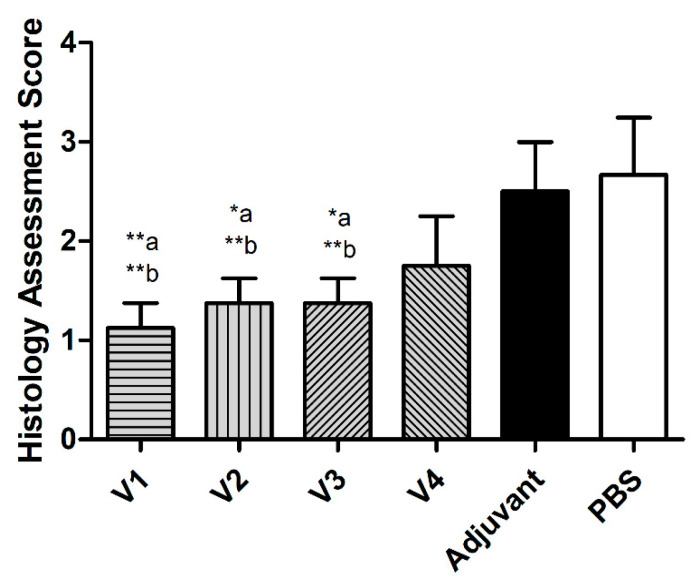
Histological scores of specimens from different vaccinated (V1, V2, V3, and V4) and unvaccinated (adjuvant and PBS) groups. Data are presented as mean ± SD and statistically analyzed by one way ANOVA, followed by the Tukey–Kramer post hoc test. Statistical significance was considered when * *p* ≤ 0.05 and ** *p* ≤ 0.01. “a” vs. PBS group and “b” vs. adjuvant group.

Four gastric samples from each vaccinated, adjuvant, and PBS group were histologically evaluated. Three magnifications (20× and 40×) were examined for one representative sample from each group (Figure 4). The photomicrographs in Figure 4 demonstrate a representative area of a section from each group, showing the difference in infiltration of inflammatory cells between different groups. Histological slides of stomach tissues from all vaccinated groups (V1, V2, V3, and V4) showed normal structure of the gastric epithelial cells with less inflammation to patchy infiltration of mixed leukocytes extending in the mucosa and submucosa. At the microscopic level, the V1 group demonstrated few patchy infiltrations of mixed leukocytes in the mucosa and submucosa, while the V2 and V3 groups showed a similar degree of patchy infiltration with granulocytes observed in focal areas in the mucosa and submucosa. The V4 group demonstrated more infiltration of inflammatory cells compared to the other vaccinated groups. The general histological scores of the vaccinated groups ranged between 1 and 1.9. In contrast, the histological slides of the gastric tissues from mice receiving the adjuvant and PBS without prior vaccination demonstrated marked increase in leukocytes with multifocal infiltration, along with multiple areas of mixed leukocytes, as compared to the vaccinated groups. The histological scores of the adjuvant and PBS groups ranged between 2 and 3 (Figure 5).

## 3. Discussion

The growing resistance rates of *H. pylori* infection have been reported to all prescribed antibiotics globally. Therefore, the only way to manage this infection and prevent associated gastroduodenal diseases would be a prophylactic vaccine, despite the extremely challenging obstacles to developing an effective vaccine [33,35]. In the present study, the antigenic proteins (CagA, VacA, and SabA) were selected using an immunoinformatic approach to shortlist the potential conserved, non-human homologous, extracellular, or outer membrane proteins. This was followed by an epitope-based analysis to identify potential B-cell-based, surface-exposed, and T-cell epitope candidates. The peptides were used to immunize BALB/c mice subcutaneously, followed by immunization challenging with *H. pylori.* The inflammatory and immunological response was evaluated biochemically by measuring the levels of IgG, IgA, IL4, and IL17 in the challenged vaccinated mice, two weeks following the *H. pylori* infection. In addition, a histopathological examination was performed to detect the degree of local inflammation on gastric tissues of the vaccinated BALB/c mice. 

The selection of proteins that match the required criteria for vaccine development is crucial. That said, we have included the CagA oncoprotein that is associated with risk of cancer [36,37,38]. The distinct effects of the VacA toxin and its effects on various cells have been reported in several studies [39,40]. Furthermore, the adherence mechanisms of SabA allow *H. pylori* to anchor in the gastric mucosa and begin the colonization process [20,41]. Recent reverse vaccinology studies recommended that these proteins be validated in both in vitro and in vivo immunological studies [23,42]. The present study concurs with a study conducted by Urrutia-Baca et. al, which reported the selection of CagA, VacA, and SabA, along with eight other proteins to produce a strong humoral and cellular immune response for *H. pylori* prevention [42]. Additionally, our findings confirmed Naz et al. in silico prioritization of VacA and SabA epitopes as a potential candidate vaccine against *H. pylori* infection [11]. Furthermore, our study successfully investigated the effect of four epitopes in a challenge protocol in BALB/c mice using a subcutaneous route of administration for the first time. Many articles have been using a similar protocol to test these vaccines in BALB/c mice, where they tested the reaction and the immunity of these mice, alongside the prophylactic and therapeutic effectiveness of the vaccines. However, different administration routes for the *H. pylori* vaccine, ranging from oral to intramuscular and subcutaneous, were proposed [43,44,45,46]. 

In the current study, the serum levels of IgG and IgA were determined in serum, given that they possess a vital role in the endogenous defense strategy against *H. pylori* [25]. Serum levels of IgG were markedly increased in SabA-immunized animals, while IgA serum levels showed a trend of increment in SabA-immunized animals, but the difference from the adjuvant and PBS groups was not significant. The immunological response to the four antigen candidates was further studied by measuring the IL-4 and IL-17 in BALB/c mice serum. The expression of the well-known inflammatory cytokine (IL-17) is positively correlated with *H. pylori* infection, particularly in gastric mucosa [26]. Notably, the inflammatory response following the injection of CagA and VacA into the gastric mucosa involves nuclear factor κB (NF-kB)-mediated IL-17/IL-8 production [27,28]. Our results also reported that the increase in IL-17 levels after challenging the animals with *H. pylori* infection would be higher in the cases of CagA, VacA, and SabA pre-immunization. Assessment of immunological responses in the present study also involved determination of IL-4 serum levels. IL-4 inhibits the inflammatory action in pathological conditions such as *H. pylori*, which triggers its plasma and gastric levels [29,47]. Conversely, some studies advocated that IL-4 has no effect on the prompted blood lymphocytes during *H. pylori* infection [30,31]. In the present study, we reported that immunization of the BALB/c mice with *H. pylori*-related peptides augments the IL-4 production after *H. pylori* infection challenge. These biochemical and immunological findings are in harmony with the histopathological investigation. The tested peptides, including CagA^1^, CagA^2^, and SabA, markedly alleviated the inflammatory patches and exudates in stomach tissues, suggesting lower necrotic response to the inflammatory environment created by the *H. pylori* challenge. Additionally, the serum values of IL-4 and IL-17 were compared to their respective control groups (PBS and Adjuvant), which is a valid way to present their induction or inhibition. Several studies determined the two ILs and displayed a comparable similar concentration range [46,48], or an even higher IL-17 concentration range, compared to IL-4 [49], despite reporting the protective effects of their vaccines. Nevertheless, we constructed a ratio between the serum levels of the anti-inflammatory cytokine (IL-4) and the pro-inflammatory cytokine (IL-17) for better demonstration of the inflammatory response in relation to different tested vaccines. Our results showed that the IL-4/IL-17 ratio favors the anti-inflammatory response of the vaccines, particularly SabA, which also comes in agreement with histopathological observations in Figure 4 and Figure 5.

The identification of adjuvants that can potentialize the specific immune response is still a major challenge in the development of an *H. pylori* vaccine. Several adjuvants have been tested in recent studies to develop vaccines against *H. pylori* [34]. In our study, we used four new epitopes and Freunde’s adjuvant to induce immunization against *H. pylori* infection in BALB/c mice. The strategy of testing an individual-epitope-based vaccine is crucial to evaluate the potentiality of each epitope as a valid vaccine before assessing the combinations. In our study, the four tested epitopes showed a favorable immunological response, especially the SabA epitope. Consequently, these results placed a spotlight on the SabA epitope, which will be prioritized in future research as part of a combined vaccine. The limitations of our study include inadequate data regarding the specific T-cell proliferation, and the assessment of *H. pylori* colonization load in gastric mucosa. Our further research will investigate the preventative and therapeutic effect of multivalent epitopes in order to study the effect of a combined vaccine against *H. pylori* infection. Only efficient vaccination would be able to prevent gastric cancer on a population-based level, and we could have an approved preventive vaccine against the *H. pylori* already if the companies spent more on vaccine development. Therefore, every year, promising approaches and recapitulate efforts that have been made to develop efficient vaccine strategies are highly appreciated [50,51].

## 4. Materials and Methods

### 4.1. Bacterial Strains and Culture Conditions

The genome sequenced *H. pylori* strain 26695 (ATCC 700392) was used in the study. The bacteria were sub-cultured on a Columbia agar base containing 10% horse serum and dent supplement (Oxoid, UK) for 72 h at 37 °C, under microaerophilic conditions (5% O_2_, 10% CO_2_, and 85% N_2_ at 95% humidity), using an anerobic jar and Campygen GasPak (Oxoid, UK). To prepare the bacterial inoculum for use in the animal model, an isolated colony was suspended into Trypticase Soya broth (Oxoid, UK), cultured for 7 h under microaerophilic conditions, and adjusted to approximately 10^7^ Colony forming unit (CFU) using (DensiCHEK^TM^ Plus, Biomerieux, Durham, NC, USA) [52].

### 4.2. Screening H. pylori Proteome to Identify Potential Protective Antigens

**Figure 6:**
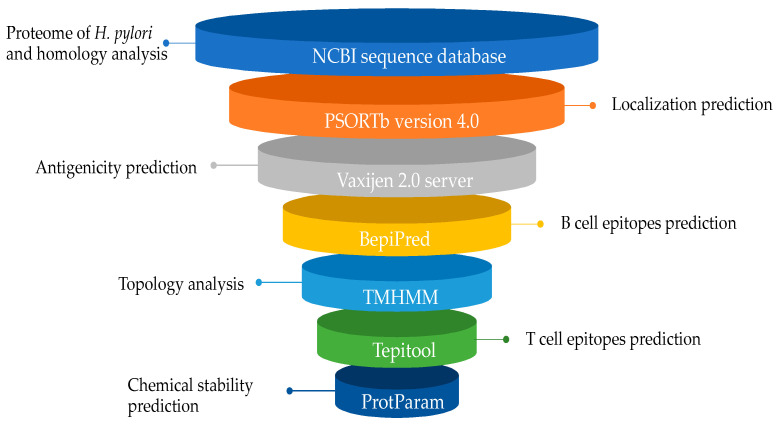
The main software used in the immunoinformatic analysis approach to shortlist the potential extracellular/outer membrane proteins, followed by an epitope-based analysis to predict antigenicity and to identify potential B-cell and T-cell epitope candidates.

The approach of immunoinformatic analysis is summarized in Figure 6. 

The complete proteome of *H. pylori* strain 26695 was retrieved from the NCBI sequence database in FASTA format. Homology analysis to human proteome was performed, using the NCBI BLASTp tool to select the non-homologous proteins (https://blast.ncbi.nlm.nih.gov (accessed on 31 May 2020) [53].

The subcellular localization of the retrieved proteins was predicted, using the cellular localization prediction tool PSORTb version 4.0, available at https://db.psort.org/ (accessed on 31 May 2020), with a cut-off score of >9.5. Proteins with localizations other than extracellular and the outer membrane were excluded [54].

The antigenicity of the retrieved proteins was predicted using Vaxijen 2.0 server for antigenicity prediction (http://www.ddg-pharmfac.net/vaxijen/VaxiJen/VaxiJen.html (accessed on 25 June 2020)). A threshold of >0.4 was used as an antigenicity value [55]. Antigenic proteins retained after the prioritization steps were used in an epitope-based analysis.

The BepiPred server was utilized to determine the antibody specific B cell epitopes (https://services.healthtech.dtu.dk/service.php?BepiPred-2.0 (accessed on 29 June 2020)); only amino acids with score of >1.3 were considered for downstream analysis [56]. The selected B-cell epitopes were then subjected to membrane topology analysis in order to determine their exposed topology by TMHMM (https://services.healthtech.dtu.dk/service.php?TMHMM-2.0 (accessed on 29 June 2020)) [57].

The T-cell epitopes that bind many MHC alleles were predicted from the shortlisted B-cell epitopes with exposed surfaces, using Tepitool http://tools.iedb.org/tepitool/ (accessed on 2 July 2020). Finally, the chemical stability of the B-cell derived-T-cell epitopes were checked using ProtParam Expacy (https://web.expasy.org/protparam/ (accessed on 9 July 2020)) [58,59].

### 4.3. Animal Model and Immunization Procedure

The study was approved by the institutional review board (IRB) at Imam Abdulrahman bin Faisal University (IAU), IRB-2019-02-315, with an approval date of 13 November 2019. Forty female BALB/c mice, approximately 5–6 weeks old (14–25 g), were provided by the animal house at the Institute for Research and Medical Consultations (IAU). They were kept in a pathogen- and stress-free environment at 24 °C, under a light–dark cycle. Water and food were allowed throughout the study. The pre-customed 4 antigenic peptides, including CagA^1^, CagA^2^, VacA, and SabA, were chemically synthesized by the Beijing Genome institute (BGI, Shenzhen, China) and were validated using HPLC and mass spectroscopy analysis.

The mice were divided into five groups (*n* = 7). Four groups were immunized by subcutaneous injection with 200 μg of the selected peptides (one per each group, purity 96%) emulsified with Freunde’s adjuvant (200 μL). Immunization was performed four times at 6 weeks, 7 weeks, 8 weeks, and 9 weeks old. The peptide, dissolved in Phosphate Buffer Saline (PBS), was emulsified with an equal volume of complete Freunde’s adjuvant for the first dose only, then with incomplete Freunde’s adjuvant for the following doses. The last booster vaccination was given without adjuvant. The fifth group received only the used adjuvant. One negative control group (*n* = 5) was given PBS. Two weeks after the last immunization, mice were challenged with *H. pylori* orally administered through gastric gavage at 11 weeks old, at a one-day interval. Finally, at 14 weeks old, mice were sacrificed by a high dose of sevoflurane inhalational (Sevorane, Aesica Queenborough Ltd., Queenborough, Kent, UK) using a Sigma Delta Vaporizer (Penlon Ltd., Abingdon, UK). The whole blood was collected using cardiac puncture, and the stomach tissues were removed aseptically for further analysis [44]. 

### 4.4. Immunological Analysis

Detection of antibodies and cytokines by Enzyme Linked Immunosorbent Assay (ELISA)kits: The whole blood was collected and centrifuged at 10,000× *g* for 10 min, and the sera were stored at −20 °C until used. Specific HRP-conjugated anti-mouse IgG and IgA as part of ELISA kits (ab151276 and ab157717, Abcam, Waltham, MA, USA) were used for the quantitative measurement of specific mouse IgG and IgA concentrations in the serum. IL-4 and IL-17 Mouse ELISA kit (ab100710 and ab100702, Abcam, Waltham, MA, USA) were used for the evaluation of cytokine production using ELISA kits; according to the manufacturer’s instructions, the absorbance was measured at 450 nm using a microplate reader (Bio-Rad xMark^TM^ Microplate Spectrophotometer, Hercules, CA, USA).

### 4.5. Histopathology Analysis

The stomach was dissected and cut longitudinally into sections. The stomach tissues were stored in a fixation solution consisting of 10% formalin for histopathological evaluation. The stomach specimens were then embedded in paraffin blocks and sectioned at 5 μm slides using a microtome. The tissue sections were stained with hematoxylin and eosin (H&E) and inspected by a histopathologist in a blinded manner using a microscope (Nikon Eclipse Ts2R, Nikon Instruments Inc., Melville, NY, USA). Each slide was assessed for the degree of gastric inflammation based on the infiltration of inflammatory cells, including lymphocytes, plasma cells, and neutrophils [43]. Sections were scored from 0 to 3 using the following criteria: (0) no inflammatory infiltrations, (1) mild patchy infiltration of inflammatory cells and exudates, (2) moderate multifocal infiltration of inflammatory cells and exudates, and (3) severe multifocal infiltration of inflammatory cells and exudates.

### 4.6. Statistical Analysis

Numerical data were presented as mean ± SD. One-way ANOVA, followed by the Tukey–Kramer post hoc test, was used to analyze data by immunological and histological analysis. Differences were considered significant at *p* < 0.05. GraphPad Prism (version 5) software was utilized for statistical analysis and graphing (GraphPad Software, Inc., La Jolla, CA, USA).

## 5. Conclusions

In the present study, antigenic proteins (CagA, VacA, and SabA) antigenic epitopes have been prioritized as potential vaccine candidates. The following step would include combining these epitopes with chitosan adjuvant in BALB/c mice in order to develop sufficiently protective humoral and cellular responses to the infection. The promising results of our study could be beneficial in the development of a peptide vaccine.

## Figures and Tables

**Table 1:** A total of four antigenic peptides were prioritized as potential vaccine candidates against *H. pylori* (CagA^1^ and CagA^2^ antigenic epitopes in addition to putative outer membrane protein (SabA), and vacuolating cytotoxin (VacA).

Code	Protein	Subcellular Localization	Peptide Sequence	NCBI Accession Number	MHC Binding Alleles	VaxiJen Results	BepiPred Results
V1	CagA^1^	Extracellular	GLGGVGQAA	WP_000180747.1	34	1.2	1.06
V2	CagA^2^	Extracellular	KLKDSTKKN	WP_000180747.1	26	1.1	1.04
V3	SabA	Outer Membrane	YQINPEQQS	WP_010875534.1	41	1.4	1.04
V4	VacA	Outer membrane	YNHLGSTNF	WP_000405496.1	30	0.8	1.06

## Data Availability

Data and materials have been provided in the main manuscript, where necessary additional information of the study can be made available from the corresponding author upon reasonable request.

## Supplementary Materials

The following supporting information can be downloaded at https://www.mdpi.com/article/10.3390/ijms232112824/s1.
